# Effects of climbing- and resistance-training on climbing-specific performance: a systematic review and meta-analysis

**DOI:** 10.5114/biolsport.2023.113295

**Published:** 2022-02-18

**Authors:** Nicolay Stien, Amund Riiser, Matthew P. Shaw, Atle H. Saeterbakken, Vidar Andersen

**Affiliations:** 1 Department of Sport, Food and Natural Sciences, Western Norway University of Applied Sciences, Sogndal, Norway

**Keywords:** Exercise, Strength, Skill, Testing, Climbers

## Abstract

The objective of this systematic review and meta-analysis was to examine the effects of climbing and climbing-and-resistance-training on climbing performance, and strength and endurance tests. We systematically searched three databases (SPORTDiscus, SCOPUS, and PubMed) for records published until January 2021. The search was limited to randomized-controlled trials using active climbers and measuring climbing performance or performance in climbing-specific tests. Data from the meta-analysis are presented as standardized difference in mean (SDM) with 95% confidence intervals (95% CI). Eleven studies are included in the systematic review and five studies compared training to a control group and could be meta-analyzed. The overall meta-analysis displayed an improvement in climbing-related test performance following climbing-specific resistance training compared to only climbing (SDM = 0.57, 95%CI = 0.24–0.91). Further analyses revealed that finger strength (SDM = 0.41, 95%CI 0.03–0.80), rate of force development (SDM = 0.91, 95%CI = 0.21–1.61), and forearm endurance (SDM = 1.23, 95%CI = 0.69–1.77) were improved by resistance-training of the finger flexors compared to climbing training. The systematic review showed that climbing performance may be improved by specific resistance-training or interval-style bouldering. However, resistance-training of the finger flexors showed no improvements in strength or endurance in climbing-specific tests. The available evidence suggests that resistance-training may be more effective than just climbing-training for improving performance outcomes. Importantly, interventional studies including climbers is limited and more research is needed to confirm these findings.

## INTRODUCTION

Rock climbing has gained increased attention in the past decade and was included in the Olympic games for the first time in 2021. In addition, a growing body of scientific literature is focusing on the physiological demands of the sport, as well as on the relationship between climbing performance and muscular strength and endurance [1]. Competitive climbing consists of three disciplines (speed climbing, lead climbing and bouldering) which differ in their respective physiological demands [2–5]. Of the three, lead climbing and bouldering are the two most practiced and researched disciplines [1]. While bouldering is performed on lower walls (< 6 meters) and often consists of few, but highly explosive and difficult moves [6], lead climbing is performed on high walls (10–30 meters) and usually consists of 20 to 50 moves with repeated, sub-maximal force generation (often referred to as endurance). Climbing performance is quantified using several different difficulty scales, depending on geographical location and discipline. Recently, however, most researchers have adopted the 1–32 numerical scale proposed by the International Rock Climbing Research Association (IRCRA) [7], making comparisons across studies possible.

Whereas climbing performance is challenging to measure reliably, assessment of the factors that may predict climbing performance is more generally applied. In general, performance in the sport of climbing relies on a complex interaction of physiological factors such as flexibility [8, 9], strength [5, 10–15], and endurance [16, 17]. Moreover, the physiological demands of climbing are influenced by factors such as the steepness of the route, the style of climbing, distance between holds, hold size, and the overall difficulty of the climb [18, 19]. While flexibility is challenging to measure [20] and has not yet received much scientific attention, strength in the finger, arm, shoulder and back muscles is relatively easy to measure in a standardized manner and has been identified as an important determinant of performance in this sport [5, 10–15]. In addition, muscular endurance assessed by using sub-maximal, intermittent contractions of the finger flexors has been shown to be related to climbing ability [16, 17].

The physiological demands of rock climbing, and the characteristics of climbers performing on different levels, have been described in previous systematic [1] and narrative reviews [21–23]. To the best of our knowledge, however, no systematic literature reviews or meta-analyses on the effect of training on climbing performance and climbing-related factors have been performed. As the training and measurement techniques vary between studies, a systematic appraisal of the current knowledge could assist researchers and athletes in the selection of prospective training and research designs. Thus, the objective of the current systematic review and meta-analysis was to assess and compare the effects of climbing- and resistance-training on climbing performance and performance in sport-specific strength-and-endurance-tests.

## MATERIALS AND METHODS

### Literature search

**FIG. 1:**
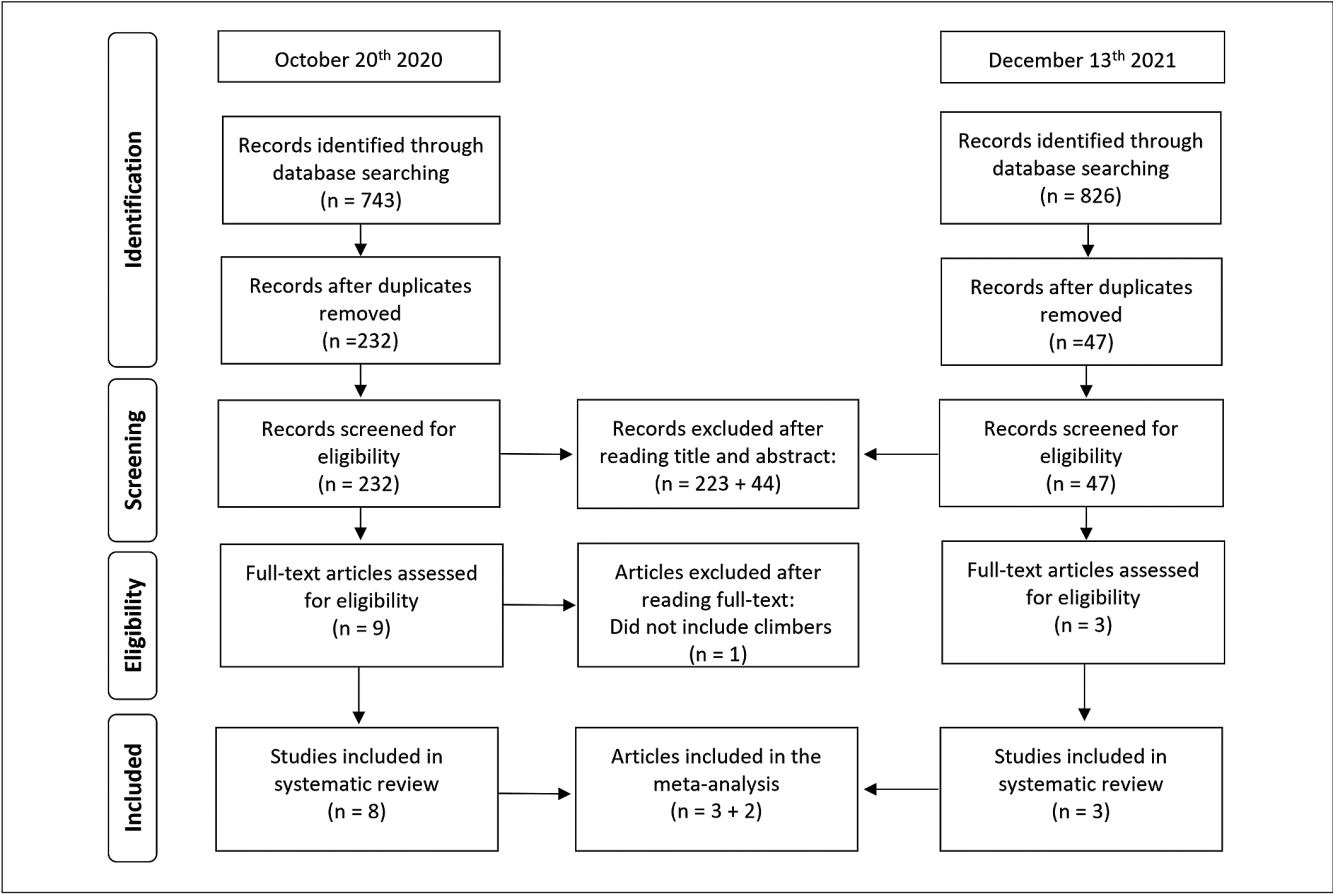
Flow chart showing the study selection procedure.

The study complies with the Preferred Reporting Items for Systematic Reviews and Mata Analyses (PRISMA) [24]. We systematically searched for published randomized control trials (RCTs) examining the longitudinal effects of climbing- and resistance-training on climbing performance or performance in sport-specific tests on October 20^th^, 2020. Peer-reviewed articles published in English were identified from three electronic databases: SPORTDiscus, SCOPUS, and PubMed. The following search terms were used: (“rock climb*” OR “sport climb*” OR “lead climb*” OR “climbers” OR “boulder*”) AND (“finger strength” OR “finger endurance” OR “forearm strength” OR “forearm endurance” OR “grip strength” OR “crimp” OR “finger flexor*” OR “training” OR “fingerboard” OR “hangboard”). The search identified 743 records (SPORTDiscus: 231; SCOPUS: 293; PubMed: 219). The search was repeated in the same databases on December 13^th^, 2021, to identify articles published after the original search. Any previously identified records were removed as duplicates. This search identified three new records published in 2021 [25–27]. All identified records were imported to EndNoteX9 and merged into one valid library to allow for removal of duplicate records [28]. After elimination of duplicates, 279 records remained (Figure 1).

### Inclusion criteria and selection process

Three authors (NS, VA and AHS) independently assessed the titles and abstracts of the studies for eligibility. In case of disagreement, subsequent consensus by discussion was reached. We included only RCTs involving active climbers of any discipline and performance level examining the effect of climbing- or resistance-training on climbing performance or climbing related physical performance such as static and dynamic finger and core strength.

### Methodological quality

The 11-item Physiotherapy Evidence Database (PEDro) scale was used to rate the methodological quality and risk of bias of the included studies [29]. This scale has previously displayed a high validity [30]. Four authors (NS, VA AHS, and AR) assessed the methodological quality independently with subsequent consensus by discussion. Please see Table 1 for an overview of the items and each of the studies’ individual score. Of the 11 items, the first item of the PEDro scale concerns external validity and is not included in the total score, leaving a maximal available score of 10 [31]. Studies with a total PEDro score > 6 were considered high-quality studies with low risk of bias [32].

**TABLE 1:** The methodological quality of the included studies, as assessed using the PEDro scale [29], the sample size *(n)* needed to obtain a = 0.05 & p = 0.2, and post hoc calculated power with a = 0.05 for the studies included in the meta-analyses [41].

Study	Eligibility specified	Randomization	Allocation concealed	Similar at baseline	Blinding of subjects	Blinding of therapists	Blinding of assessors	< 15% drop-out	Intention to treat	Between groups statistics	Point and variability measures	Total score	n for α = 0.05 and β = 0.2	Statistical power with α = 0.05
Hermans, et al. [35]	1	1	1	1	0	0	0	0	0	1	1	**5**	502	83.9
Levernier and Laffaye [33]	1	1	1	1	0	0	0	0	0	1	1	**5**	6	99.9
López-Rivera and González-Badillo [39]	1	1	1	1	0	0	0	0	0	1	1	**5**		
López-Rivera and González-Badillo [38]	1	1	1	1	0	0	0	0	0	1	1	**5**		
Saeterbakken, et al. [36]	1	1	1	1	0	0	0	1	0	1	0	**5**		
Philippe, et al. [40]	1	1	1	1	0	0	0	1	0	1	1	**6**		
Stien, et al. [27]	1	1	1	1	0	0	0	1	0	1	1	**6**	78	31.0
Mundry, et al. [25]	1	1	1	1	0	0	0	1	0	1	1	**6**	456	6.1
Medernach, et al. [34]	1	1	1	1	0	0	0	1	1	1	1	**7**	126	18.5
Medernach, et al. [37]	1	1	1	1	0	0	0	1	1	1	1	**7**		
Stien, et al.[26]	1	1	1	1	0	0	0	1	1	1	1	**7**		

1 = criterion is satisfied/fulfilled, 0 = criterion is not satisfied/fulfilled

### Data extraction and analysis

Data extraction was completed in accordance with the Cochrane handbook for systematic reviews of interventions [28]. NS and AR conducted data extraction of study results separately and settled discrepancy by mutual agreement. Studies were found appropriate for meta-analysis [25, 27, 33–35] if they performed any climbing- or resistance-training method in the intervention group and compared the changes to a passive (i.e., no climbing or climbing-specific training) or active (i.e., climbing- or resistance-training as usual) control group. Changes in finger strength were extracted from Levernier and Laffaye [33], and Medernach et al., [34], and Stien et al. [27] while changes in dead hang duration were extracted from Medernach et al. [34] and Hermans et al. [35]. When several outcomes were presented in the same intervention, only one outcome was included in each meta-analysis. Finger strength was prioritized over rate of force development (RFD) and dead-hang duration in the main analysis. Finger strength was prioritized because 1) this was the most reported outcome in the included studies and 2) has been identified as a crucial determinant of climbing performance [11, 14]. Other parameters (e.g., bent-arm hang and climbing performance) that were only examined in one study, or were not measured or trained in comparable ways, were excluded from the meta-analysis. Studies that fulfilled the inclusion criteria but did not use a control group [26, 36–40] were excluded from the meta-analysis and included in the systematic review.

### Statistical analyses

Extracted data from individual studies were collated in Excel (Microsoft) and meta-analyses were performed in Comprehensive Meta-Analysis (CMA) V.3 (Biostat, Englewood, New Jersey, USA). The meta-analyses were performed with random effects models, and effect estimates are presented as standardized difference in mean (SDM) with 95% CI. Heterogeneity is presented as I^2^ and p-values. Potential publication bias was assessed by funnel plot and Begg and Mazumdar rank correlation test. Post hoc power calculations with α = 0.05 and adequacy of sample size in each of the included studies was assess by calculation of the sample size required for the effect in each study to obtain an alpha of 0.05 and a beta of 0.2 using an online calculator [41]. As normality is an important assumption for meta-analyses, skewness of the outcomes was assessed as baseline mean/SD and variables with a mean to ratio > 2 were considered skewed [42]. The significance level was set to p < 0.05.

## RESULTS

### Study characteristics

Finally, the same three authors read the full-texts of the remaining twelve studies and agreed to remove one due to not fulfilling the inclusion criteria of including active climbers. The reference lists of the included papers were manually searched to discover additional relevant studies. However, this method yielded no further results. The present systematic review consists of eleven published studies comprising 225 climbers (Table 2). The overall meta-analysis comprised 110 climbers from 5 studies [25,27, 33–35]. The trials compared the effect of resistance-training with climbing on performance in climbing-specific strength- and endurance-tests. Stratified analyses were performed on two studies [34, 35] investigating the effect of resistance-training on dead hang ability comprising 53 climbers, and four studies [25, 27, 33, 34] investigating the effect of finger resistance-training on finger strength comprising 80 climbers. The six studies [26, 36, 37, 39, 40, 43] not included in the meta-analyses, due to not including a control condition, comprised 115 climbers.

**TABLE 2:** Characteristics of the included studies.

	Subjects (n, sex, age)	Performance level	Intervention	Control	Outcomes
López-Rivera and González-Badillo [39]	8 m, 1 f 30.4 ± 3.9	Advanced and elite sport climbers	Fingerboard training		Finger strength: 5 s hang 15 mm edge max load, Finger endurance: half crimp dead hang 11 mm edge
		IRCRA ≤ 23	4 wk MED + 4 wk MAW		
			4 wk MAW + 4 wk MED		

Medernach, et al. [34]	23 m, 25.6 ± 4.4	Highly advanced male boulderers	Fingerboard training:	Bouldering:	Handheld Dynamometry, Dead hangs (19 mm), Intermittent finger hangs (30 mm)
		IRCRA = 23	4 wk, 3 × 150 min per wk	4 wk, 3 × 150 min per wk	

Medernach, et al. [37]	24 m, 25.2 ± 4.8	Advanced boulderers	Interval bouldering, 4 wk, 3 × 150 min per wk	Conventional bouldering, 4 wk, 3 × 150 min per wk	Intermittent hangs (30 mm), Climbing-time to exhaustion
		IRCRA = 22			

Hermans, et al. [35]	30 m/f 23.3 ± 1.9	Lower-grade and intermediate climbers	Upper-body resistance training:	Continued climbing/training as usual	Climbing performance, Dead-hang, Bent-arm hang, 12RM pull-down on machine
			10 wk, 7 exercises × 4 sets × 5RM, 2 times per wk		
		IRCRA = 8–13	10 wk, 7 exercises × 2 sets × 20RM, 2 times per wk		

Saeterbakken, et al. [36]	13 m, 6 f 27.4 ± 6.7	Elite and Advanced climbers	Trunk muscle training:		Body lock-off, Body-lift, Superman, Isometric core strength tests, Finger hang test
		IRCRA = 20.1 ± 3.1	10 wk 4 Isometric exercises, 3–4 sets × 4–10 reps, × 2 /wk		
			10 wk 4 dynamic exercises, 3–4 sets × 4–10 reps, × 2 /wk		

Levernier and Laffaye [33]	14 m, 26.1 ± 2.2	Elite and top world-ranking climbers	Isometric half-crimp strength:	Continued climbing/training as usual	Max isolated finger strength and RFD
		IRCRA > 25	4 wk, 6 exercises, 2 series of 4–6 s effort, 3 times pr wk		

López-Rivera and González-Badillo [38]	23 m, 3 f, 32.0 ± 6.2	Advanced and elite sport climbers	Fingerboard training		Finger endurance: half crimp dead hang 11 mm edge
			8 wk max weight hangs		
		Mean IRCRA = 22			
			8 wk intermittent hangs		

Philippe, et al. [40]	15 m, 8 f, 25.5 ± 6.7	Elite climbers	8 wk climbing-specific muscle endurance training		On sight climbing 17 m Overhang: 27.9° 48 moves
		IRCRA = 20.8 ± 2.0			
			8 wk climbing-specific hypertrophy training		

Stien, et al. [27]	16 m, 30.3 ± 7.4	Advanced and elite climbers	5 wk campus board training	Continued climbing/training as usual	Bouldering, campus board performance, isometric
			Volume divided over 2 weekly sessions		pull-up strength and RFD (jug and 23 mm rung), arm circumference
		IRCRA = 21.2 ± 2.7	Volume divided over 4 weekly sessions		

Stien, et al. [26]	11 m, 3 f, 27.3 ± 5.3	Intermediate and advanced climbers,	Climbing training		Lead climbing, bouldering, forearm endurance (intermittent test), Max isolated finger strength (23 mm rung), isometric pull-up strength and RFD (jug and 23 mm rung)
			5 wk, three weekly sessions (2 lead and 1 boulder)		
		IRCRA = 16.3 ± 2.4			
			5 wk, three weekly sessions (1 lead and 2 boulder)		

Mundry, et al. [25]	15 m, 12 f, 24.7 ± 3.5	Intermediate to advanced	Fingerboard training	Continued climbing/training as usual	Grip strength in 7 different pinch grip positions and handgrip dynamometry
		IRCRA = 14 ± 4	8 wk MED		
			8 wk MAW		

m = males, f = females, n = number, IRCRA = International Rock Climbing Research Association grde reported in the studies, wk = week, s = seconds, mm = millimeters, RFD = rate of force development, MED = minimum edge depth, MAW = maximal added weight.

A heterogeneity was observed for the study samples of the studies included in this systematic review and meta-analysis. Specifically, the performance levels ranged from lower-grade climbers [35] to elite and top internationally-ranked athletes [33]. This challenges the ability of this systematic review and meta-analysis to provide recommendations for specific performance levels. However, the variability in performance levels within studies was generally low. Unfortunately, some studies [35, 36, 40] failed to specify the predominant discipline of the participants. Current recommendations [7] suggest that studies should report how climbers classify their participation in the sport (e.g., sport climber, boulderer, speed climber, etc.) to allow for more detailed interpretation of the findings. It is further recommended that studies report the percentage of time devoted to each discipline [7], which none of the studies included in the present review did.

### Quality of the studies

Five studies [33, 35, 36, 38, 39] fulfilled five items on the PEDro scale and the remaining studies fulfilled 6 [25, 27, 40] or 7 items [26, 34,37]. All studies had eligibility specified, concealed allocation, randomized the climbers into groups and the groups were similar at baseline (Table 1). None of the studies blinded the allocation of the climbers to the investigators and assessors, or the climbers themselves. Moreover, none of the studies with dropouts conducted intention-to-treat analyses.

### Results from the meta-analysis

**FIG. 2:**
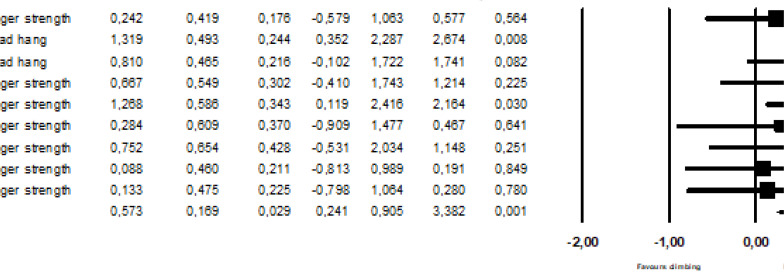
Main analysis of the effects of climbing specific resistance-training on finger strength and forearm endurance.

**Supplementary figure 1:**
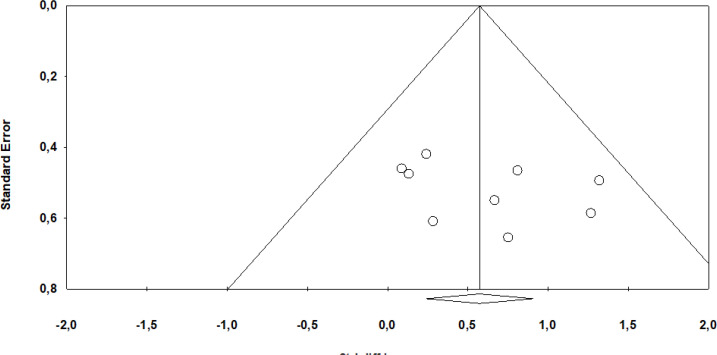
Funnel plot of the effect of finger resistance-training on finger strength. The plot indicates no proclivity for publishing studies that found a significant effect of training.

In an overall analysis combining nine trials from five studies comparing the effect of resistance-training and a control condition [25, 27, 33–35], resistance-training improved performance in climbing-specific tests (dead-hang duration or finger strength) compared to regular climbing-training (SDM = 0.57, 95%CI = 0.24–0.91; Figure 2). The included studies were not heterogeneous (I^2^ = 0%, p = 0.47). The SDM for the studies included in the stratified analysis were not heterogeneous (I^2^ = 0%, p > 0.72). The funnel plot and the Begg and Mazumdar Rank Correlation Test (p = 0.174) did not indicate publication bias (please see Supplementary Figure 1) and the classic fail-safe N was 20, indicating that there must be 20 unpublished studies to bring the effect of finger resistance training on finger strength to a p > 0.05. One of the five studies included in the meta-analysis included an adequate number of participants to obtain an α = 0.05 and a β = 0.2 and two of the studies had a statistical power > 80% (Table 1). None of the baseline performance data were skewed.

### Meta-analysis of finger strength

**FIG. 3:**
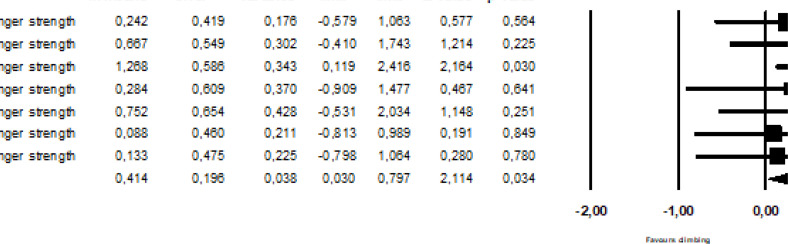
The effect of finger resistance-training on finger strength.

The meta-analysis of the effects of finger resistance-training included seven trials from four studies [25, 27, 33, 34]. All studies compared fingerboard training with a control group that continued climbing training as usual, and tested finger strength using a half-crimp grip on a climbing-specific hold, either in isolation or with an unconstrained elbow. The analyses revealed that finger strength was improved by specific, isometric finger resistance-training compared to climbing training (SDM = 0.41, 95%CI = 0.03–0.80) (Figure 3).

### Meta-analysis of rate of force development

**FIG. 4:**
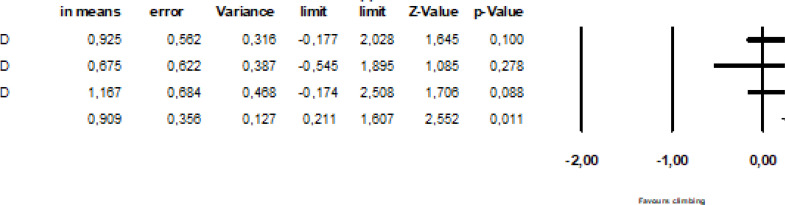
The effect of finger resistance-training on rate of force development.

The effect of finger training on rate RFD was assessed in two studies including three trials [27, 33]. The studies compared finger- or campus-board training with a control group that continued climbing training as usual, and tested RFD using a hand dynamometer (half crimp) and isometric pullup on a 23 mm rung. RFD was improved by finger strength training compared to climbing as usual (SDM = 0.91, 95%CI = 0.21–1.61; Figure 4). The SDM for the studies included in the stratified analysis were not heterogeneous (I^2^ = 0%, p > 0.87).

### Meta-analysis of dead-hang endurance

For dead-hang endurance, a stratified analysis of three trials from two studies [34, 35] was performed. The training included isolated, isometric resistance-training on a climbing-specific hold [34], or forearm curls using dumbbells [35]. Dead-hang duration assessed on either a 19 mm [34] or 25 mm [35] deep rung was improved by resistance-training of the fingers and forearms compared to climbing training (SDM = 1.23, 95%CI = 0.69–1.77).

### Results not included in the meta-analysis

Six of the included studies [26, 36, 37, 39, 40, 43] could not be included in the meta-analysis as they did not include a control group. The findings of these studies are presented below. Further, some results from the studies included in the meta-analysis [27, 33, 35] could not be analysed because the outcomes are presented in only one study and are, therefore, also presented here.

### Climbing performance

Five of the studies included in this review measured changes in climbing performance [27, 35, 37, 40]. Although a control group was included in two of these studies [27, 35], the methodological differences (training- and testing-procedures) made it unfeasible to include the results in the meta-analyses. Hermans et al. [35] reported non-significant tendencies toward improved lead climbing performance following both low-resistance-high-repetitions (12.0%, p = 0.088) and high-resistance-few-repetitions (11.3%, p = 0.090) upper body resistance-training (e.g., pull-downs, biceps curl, and forearm curl). Neither training modality was superior to the other (p = 0.420–0.950). Philippe et al. [40] compared the effects of climbing-specific muscular endurance training (combination of hard and easy lead climbing) and muscular hypertrophy training (bouldering, campus board, and hard lead climbing). Both groups improved on-sight lead climbing performance (p < 0.001), but the improvements were not different between the groups (p = 0.542–0.955). Campus board training was also implemented in a study by Stien and colleagues [27] in which two training frequencies (two and four weekly sessions) were compared to an active control group. No significant difference between the two training groups was found, but only the group that trained two times per week improved bouldering performance more than the control group. Moreover, Medernach et al. [37] reported significantly greater improvements (p = 0.004) in climbing time to exhaustion following interval bouldering (36.2 ± 14.1 seconds, p < 0.001), compared to conventional bouldering (6.1 ± 19.3 seconds, p = 0.298). Finally, Stien and colleagues [26] displayed no within- or between-groups differences following five weeks of lead- or boulder climbing training.

### Finger strength

López-Rivera and González-Badillo [39] reported no significant improvements in finger grip strength following four weeks isolated finger resistance-training (2.1–9.6%). The non-significant change in force was significantly greater following dead hang training using maximal external load on a deep rung compared to dead hang training using no external load and the shallowest rung possible in the training. Moreover, Stien et al. [26] reported an increase in isolated finger strength following five weeks of bouldering training (ES = 0.35, p = 0.030), but not lead-climbing training. Importantly, the two studies are difficult to compare due to differences in both training and testing procedures.

### Forearm endurance

López-Riviera and González-Badillo [43] demonstrated that forearm endurance improved after four weeks of implementing intermittent dead hangs (25.2%, p = 0.004), and not after maximal weighted dead hangs or a combination of the two. López-Riviera and GonzálezBadillo [39] found no change in dead hang endurance following minimal edge or maximal weighted dead hangs, whereas Medernach et al. [37] observed improved intermittent finger hang time following interval bouldering (27.3 ± 18.4 seconds, p < 0.001), but not conventional bouldering (4.9 ± 11.5 seconds, p = 0.168). The intermittent finger hang time improvement was significantly greater following interval bouldering (p < 0.001). Similarly, Stien et al. [26] found no changes in intermittent forearm endurance following conventional bouldering training, but did report an increased forearm endurance following lead-climbing training (ES = 0.55, p = 0.014).

## DISCUSSION

To the authors’ knowledge, this is the first systematic review and meta-analysis examining the effects of different climbing- and resistance-training interventions on climbing performance and climbing-specific muscle strength and -endurance. The main findings from this meta-analysis were that climbing-specific finger endurance was significantly improved following forearm resistance-training [35] and isolated finger training [34], with isolated finger resistance-training improving finger strength more than climbing training alone [33, 34].

### Quality scores of the included studies

Of the studies discovered in the systematic search, eleven met all the inclusion criteria. The scores on the PEDro scale for the included studies ranged from 5 to 7 (median = 6) on the 10-point scale, and five RCTs could be included in the meta-analysis. The relatively low sample size provides low statistical power, and although the sample size in this systematic review and meta-analysis is larger than in all individual studies, the results should be interpreted with caution. Moreover, the need for more high-quality studies in the field of climbing-performance is evident. Specifically, none of the studies utilized blinding of the participants or researchers, and none of the interventions with one or more drop-outs conducted intention-to-treat analyses.

### Meta-analyses

The included studies reporting on finger strength [25,27, 33, 34] examined highly trained climbers (IRCRA ≥ 18). A small-to-medium effect was observed for finger strength after a four-to-five-week finger resistance-training intervention and the improvements could be interpreted as meaningful due to the high performance level and long climbing experience (≥ 4 years) of the included climbers. Moreover, considering the short duration of the interventions, finger- and campus-board training appears to be highly effective training methods that climbers can be implemented in a short training block to emphasize finger strength before competitions. The findings could be explained by the fact that such training prioritizes the finger flexors intensely in a structured and specific training program, whereas climbing training may provide a more varied approach that also trains, and is limited by, other muscles and skills. Finally, marked improvements in finger strength following isolated resistance-training of the finger flexors may be explained by the principle of specificity [44].

Three trials from two studies including only male climbers performing on an advanced-to-international level were included in the meta-analysis of RFD [27, 33]. Stien et al. [27] investigated the effects of campus board training performed either two or four times per week with an equated volume. The authors reported that four, but not two weekly sessions improved RFD more than the control group. In the study by Levernier and colleagues [33], the authors examined the effect of fingerboard in top world-ranking climbers and found that a four-week intervention improved unilateral finger flexor RFD more than continuing climbing training as usual. Although the two studies differ in study population, training intervention, and testing methods, it should be of interest that a short training intervention (4–5 weeks) of supplemental resistance training seems able to improve RFD (which is arguably among the strongest predictors of climbing performance [14, 45]) in highly trained climbers. In fact, the studies demonstrated large effects (SMD = 0.90) for improvements in RFD compared to climbing as usual. Importantly, only a small number of highly trained, male climbers were included in the studies and the findings may not be generalizable to other populations.

The two studies reporting forearm endurance [34, 35] included in the stratified analysis comprised young climbers (age: ˜ 23–26 years) on a lower grade and intermediate level [35], and highly advanced boulderers [34]. Both studies reported improved forearm endurance following two different approaches to the training. Still, the difference in the study samples makes comparisons of the two studies difficult and challenges the validity of this analysis. The forearm endurance training in the study by Hermans et al. [35] consisted of forearm curls using a dumbbell, which is not an exercise commonly implemented among climbers and may lack specificity toward the endurance test performed on a shallow rung. One can speculate that the low performance level of the climbers allowed for a non-specific training method to produce significant improvements in the forearm endurance test. Medernach et al. [34] implemented four weeks of resistance-training of the fingers using a shallow rung which is a more specific training method, both for the dead hang endurance test and for climbing. The high specificity toward the dead hang endurance test is probably why this method proved efficient among the highly advanced boulderers included in the study following a short intervention.

### Systematic review

The trials that could not be included in the meta-analysis due to no comparison with a control group or the existence of no comparable trials, were included in the systematic review [26, 33, 35–40, 46]. Interestingly, and in contrast to the meta-analysis, most of these studies did not demonstrate significant improvements in forearm endurance or finger strength [38, 39], likely due to the elite performance level of the participants and the low sample sizes. In line with this speculation, the studies included in the meta-analysis [33–35] examined a higher number of climbers performing on a wide range of levels, which could potentially allow potential changes to be more easily detected. However, one study included in the systematic review [26] reported improved finger strength and endurance following bouldering and lead climbing, respectively. The difference could be explained by the intermediate performance level of the participants in the latter study. It should be noted that the generalizability of the individual articles included in this study is limited by the small study samples and varying performance levels and disciplines between studies. The results of both the individual studies and this systematic review must, therefore, be interpreted with caution.

The core has been described as a crucial factor for transferring force throughout the body [47] and core strength has been identified as a secondary determinant of climbing performance after shoulder-strength and -power [48]. One study examined the effect of dynamic or isometric core strength training in climbers [36] and reported improvements in climbing-specific tests (e.g., body lock-off and body-lift), but no significant between-groups differences. Importantly, climbing performance was not tested in the study. Interestingly, Muehlbauer and colleagues [49] found that MVIC of the trunk flexors improved among non-climbers following eight weeks of two weekly indoor climbing sessions. Furthermore, Hermans et al. [35] implemented general upper body resistance-training and found no changes in climbing performance following low or high numbers of repetitions using high or low loads on climbing performance. With a performance level ranging from lower-grade to intermediate, it is possible that the climbers could benefit more from specific climbing training than from general resistance-training. However, cross-sectional studies have highlighted the importance of shoulder power [48] and elbow flexor strength [50] for climbing performance. Hence, it can be speculated that implementing a similar intervention among more accomplished climbers and using a larger sample size could demonstrate positive effects on climbing performance.

### Study characteristics

Most of the intervention studies [25–27, 33, 34, 37–40] were of short duration (four to eight weeks) and the two longest studies lasted no more than ten weeks [35, 36]. Moreover, these were the only two studies that did not include climbing-specific finger resistance-training or changed the climbing routines of the participants in the intervention. Further, two of the studies that lasted eight weeks compared two groups performing very similar training programs: intermittent vs. maximal weighted dead-hangs [43] or endurance vs. hypertrophy training) [40]. The same was true for two of the studies with four-week interventions which also compared two similar training approaches: minimal edge vs. maximal weighted dead-hangs or [39] interval bouldering vs. conventional bouldering [37]. This leaves three studies [25, 33, 34] that were able to identify possible effects of climbing-specific resistance-training of the fingers compared to the effects of continuing climbing training as usual. Importantly, short interventions are not necessarily a limitation in climbing-re-search. Four weeks is a common duration of blocks in resistance training [51] and as specific finger strength and endurance training involves very high intensity training on small muscles, periodization in four-week blocks is likely a reasonable method for avoiding overuse injuries.

Regarding gender, female climbers are underrepresented in the literature and no interventional studies have included only females. Five studies included in this systematic review and meta-analysis included only males, whereas the remaining seven combined males and females. Hermans et al. [35] did not report the distribution of males and females, while the remaining studies [25, 26, 36, 38–40] had a majority of males (total: 100 males and 45 females). A difference in strength and hypertrophy between men and women with identical training background has been identified [52]. However, it has also been shown that physically active males and females respond similarly in the first weeks (up to 12 weeks) of high-intensity resistance-training programs [53, 54]. Although males and females may respond similarly to climbing-specific resistance-training, the distinct effects on female climbers are yet unknown and should be investigated in future research.

### Limitations

As the field of research examining climbing-specific resistance-training is relatively young and only very few interventional studies have been conducted, it can be speculated that comparisons of slightly different training methods are premature. At this point, interventional studies on climbers should rather identify the effects of training than compare different but relatively similar methods. For example, the effects of two [39] or three [38] highly similar finger resistance-training modalities have been compared and revealed few or no differences in effect. Hence, resources could be better spent comparing the effects to a control group, rather than to a training group performing resembling training programs. Many of the interventional studies performed on climbers comprise relatively few participants, but by performing a meta-analysis we increased the statistical power to detect differences compared to the statistical power in the individual original studies. However, low sample size in studies/comparisons included in meta-analyses, as in the present study, may introduce spare data bias in the standardized difference in mean [55]. Thus, the standardized difference in mean and its confidence intervals should be interpreted with caution. All meta-analyses indicated no heterogeneity (I^2^ = 0), but I^2^ has substantial bias when the number of studies in a meta-analysis is low and the number of studies/comparisons in our meta-analysis ranged from three to nine, and if the true heterogeneity is high the heterogeneity will be underestimated [56]. The I^2^ values in the present study should therefore also be interpreted with caution. Four out of five studies included in the meta-analysis did not include an adequate number of participants to obtain an α = 0.05 and a of β = 0.2 which indicates that the included studies were under-powered. Another limitation is that differences in performance level of the study populations (ranging from lower-grade lead climbers to highly advanced boulder climbers) challenge the comparability between studies. Furthermore, the present study is limited by the fact that only three of the included papers [34, 37, 40] received a PEDro score that met the criteria for high-quality studies (≥ 6). One inherent limitation of training studies is the difficulty in blinding of researchers and participants. However, researchers examining climbers should strive to avoid reporting bias in future studies. Finally, as only studies published in English were included, it is possible that papers written in other languages contain further information not included in this systematic review and meta-analysis.

### Recommendations for future studies

This systematic review with meta-analysis provides an overview of the current knowledge of the effects of different training approaches on climbing performance and performance in climbing-specific tests. An important finding was the scarcity of scientific, longitudinal literature. Moreover, only three interventional studies could be included in the meta-analysis, due to many studies not including a control group, but rather a secondary training intervention. Hence, it is recommended that upcoming interventional studies include a control group to explicitly target the effects of the intervention compared to not changing the training routines of climbers. Further, female climbers are underrepresented in the literature and no study has yet examined the effects of climbing or climbing-specific training among only females. Another recommendation based on the current findings is for future research to examine the long-term effects of training interventions in climbers. Thus far, no study has had a duration longer than ten weeks, with most interventions lasting from four to eight weeks. From a practical point of view, future studies should include a measure of climbing performance if possible. Although performance in climbing-specific tests is an indicator of climbing performance, the underlying aim of most research in the field is to explore how climbing performance can be improved. Importantly, this is a highly difficult measure to conduct due to the high complexity of the sport. Still, novel tools such as the Kilter board may present the possibility of including reproducible measurements of climbing performance in future research. Finally, as previously recommended [7], studies should clearly describe their study population regarding preferred discipline, weekly training and climbing volume, performance level, climbing experience and potential block periodization implemented in their regular training routines.

## CONCLUSIONS

To our best knowledge, this was the first systematic review and meta-analysis examining the longitudinal effects of climbing and climbing-specific resistance-training on climbing performance and performance in climbing-specific tests. Although the field of climbing research is still in its infancy, some conclusions can be drawn from the available literature. For climbers, it is evident that the addition of systematic resistance-training (e.g., fingerboard, campusboard, or upper-body resistance training) can yield greater improvements in climbing-specific fitness than climbing-training alone, across several performance levels. However, the scarcity of studies limits the possibility of recommending specific training methods. Hopefully, this systematic review and meta-analysis can assist researchers in designing future intervention studies focused on climbers. Specifically, there is a need for 1) studies comparing training to a control condition, 2) studies including only female climbers, and 3) studies that more clearly describe the participants to allow for more precise comparisons and discussions of findings across studies.
